# Supplementary material for: Coherent control of asymmetric spintronic terahertz emission from two-dimensional hybrid metal halides
Source: Nat Commun. 2021 Sep 30;12:5744. doi: 10.1038/s41467-021-26011-6 (PMC8484356; doi:10.1038/s41467-021-26011-6)
Supplement: Supplementary file 1 — Supplementary Information [file 41467_2021_26011_MOESM1_ESM.pdf]

--- *Supplemental Information* ---

Coherent Control of Asymmetric Spintronic Terahertz Emission from Two-Dimensional Hybrid Metal Halides

Kankan Cong[1#], Eric Vetter[2,3#], Liang Yan[3,4#], Yi Li[5,6], Qi Zhang[1§], Yuzan Xiong[5,7], Hongwei Qu[7], Richard Schaller[8], Axel Hoffmann[6,9], Alexander F. Kemper[2], Yongxin Yao[10], Jigang Wang[10], Wei You[3,4*], Haidan Wen[1,*], Wei Zhang[5,6,*], and Dali Sun[2,3*]

[1]*Advanced Photon Source, Argonne National Laboratory, Argonne, IL 60439, USA*
[2]*Department of Physics, North Carolina State University, Raleigh, NC 27695, USA*
[3]*Organic and Carbon Electronics Lab (ORaCEL), Raleigh, NC 27695, USA*
[4]*Department of Chemistry, University of North Carolina at Chapel Hill, Chapel Hill, NC 27599, USA*
[5]*Department of Physics, Oakland University, Rochester, MI 48309, USA*
[6]*Materials Science Division, Argonne National Laboratory, Argonne, IL 60439, USA*
[7]*Department of Electronic and Computer Engineering, Oakland University, Rochester, MI 48309, USA*
[8]*Center for Nanoscale Materials, Argonne National Laboratory, Argonne, IL 64039, USA*
[9]*Department of Materials Science and Engineering, University of Illinois at Urbana-Champaign, Urbana, IL 61801, USA*
[10]*Ames Laboratory and Department of Physics and Astronomy, Iowa State University, Ames, IA 50011, USA*
[§]*Present address: Department of Physics, Nanjing University, Nanjing, 210093, P. R. China*
[*]*Corresponding emails:* wyou@unc.edu, wen@anl.gov, weizhang@oakland.edu, dsun4@ncsu.edu,

Appendix:

I. **Device fabrication**
II. **2D-HMH film characterization**
III. **Spintronic THz emission in control samples**
IV. **Pump helicity dependence**
V. **THz in 3D-HMH-based THz emitter**
VI. **THz transmission**
VII. **Sample anisotropy**
VIII. **Role of SiO_2 capping layer for THz emission**
IX. **Asymmetric THz emission in other reduced-dimensional HMH-based THz emitters**
X. **Pump fluence dependence**
XI. **Summary of control experiments**

I. Device fabrication

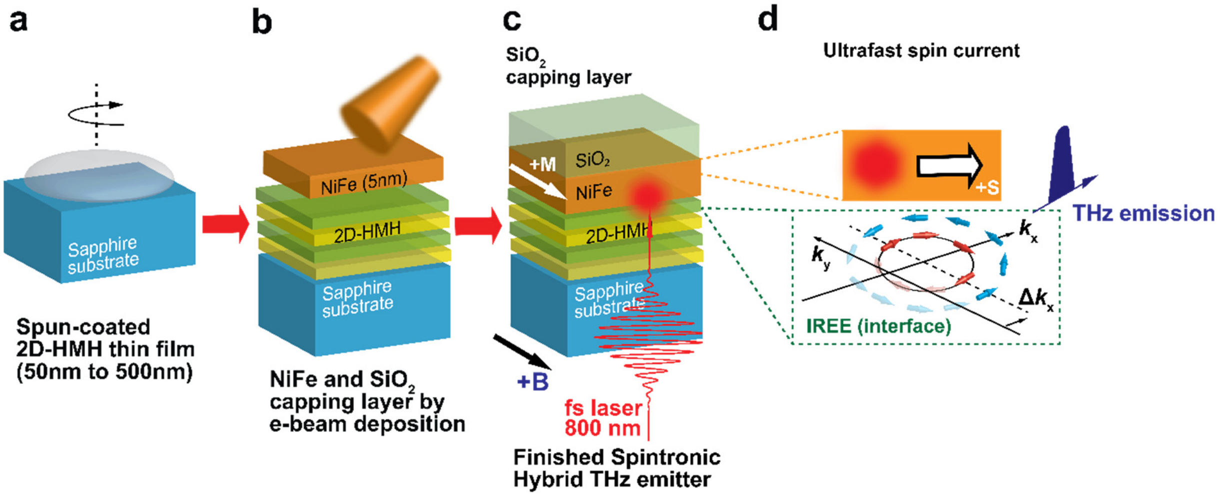

Figure S1. Schematic representation of sample preparation process where (a) the 2D-HMH thin film is first spin coated onto a 5 x 5 mm sapphire substrate followed by (b) e-beam deposition of the 4 x 4 mm NiFe thin film and subsequent 5 x 5 mm SiO_2 capping layer. (c) and (d) Schematic of the spintronic THz emission process whereby an incident 800 nm fs laser pulse causes ultrafast demagnetization of the NiFe layer followed by ultrafast spin current injection into the NiFe/2D-HMH Rashba interface where spin-to-charge conversion transforms the injected spin current into a transient THz electric field.

II. 2D-HMH Film Characterization

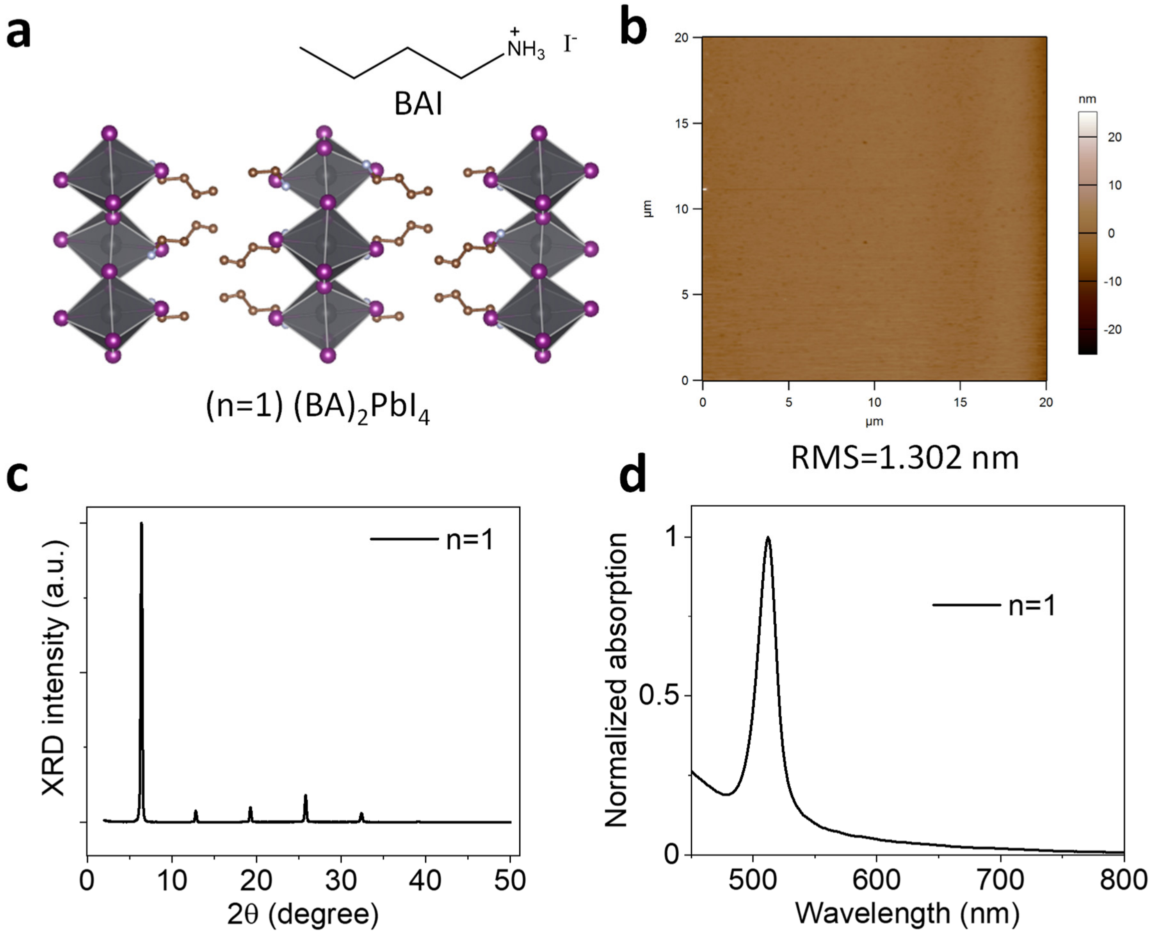

Figure S2. **a**, Schematic illustration of the 2D-HMH crystal structure where butylammonium cations are layered between the lead halide framework. **b**, Representative AFM scan of the spin coated 2D-HMH film where the RMS roughness is approximately 1.3 nm. **c**, X-Ray diffraction pattern obtained for the n = 1 2D-HMH spin coated film and (**d**) its corresponding UV-Vis absorption spectrum.

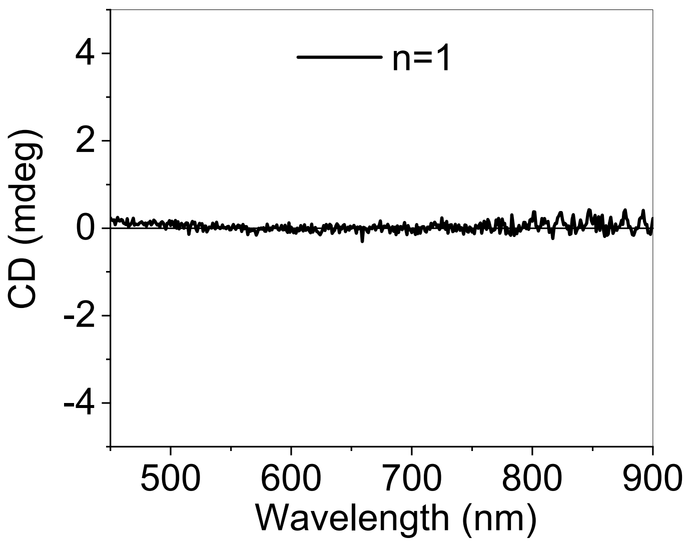

Figure S3. Circular dichroism spectrum for (n = 1) 2D-HMH spin coated perovskite film, showing the absence of chirality in 2D-HMH without chiral organic cations.

III. Spintronic THz emission in control samples

Comparison of the THz spectra in the NiFe/Pt and 2D-HMH/NiFe device

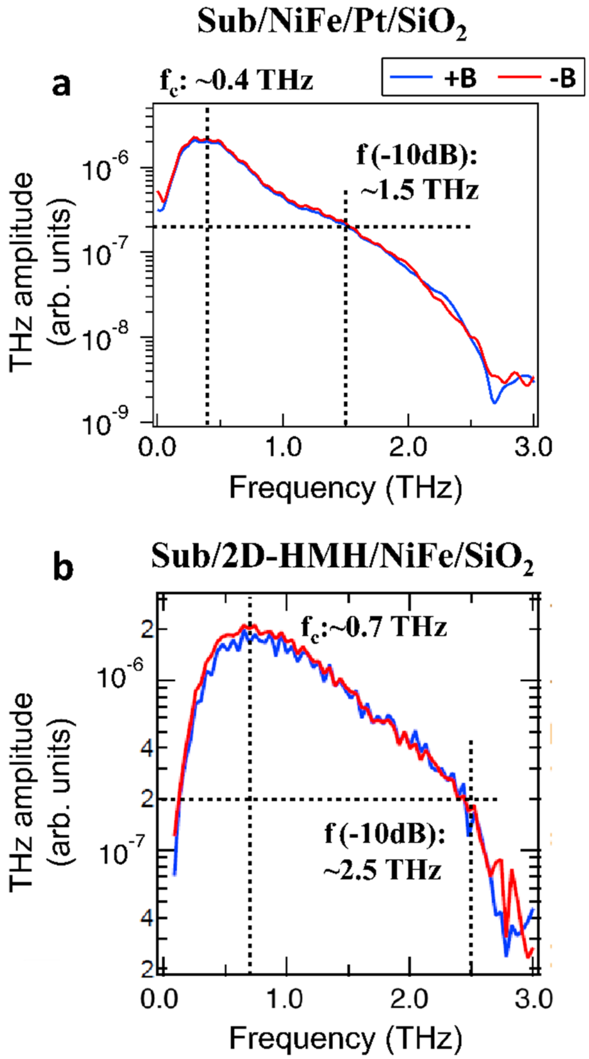

Figure S4. Comparison of the THz spectra in the NiFe/Pt (**a**) and 2D-HMH/NiFe device (**b**), respectively.

The THz spectra obtained from the 2D-HMH/NiFe device indeed exhibit a distinct frequency domain in contrast to that in the NiFe/Pt device. We found the maximum THz frequency from the NiFe/Pt device is located at fc= ~0.4 THz, whereas the one from the 2D-HMH/Pt device is at 0.7 THz. The -10dB point from the NiFe/Pt device is located at 1.5 THz while the THz bandwidth from the 2D-HMH/NiFe device is broader (up to 2.5 THz).

IV. Pump helicity dependence

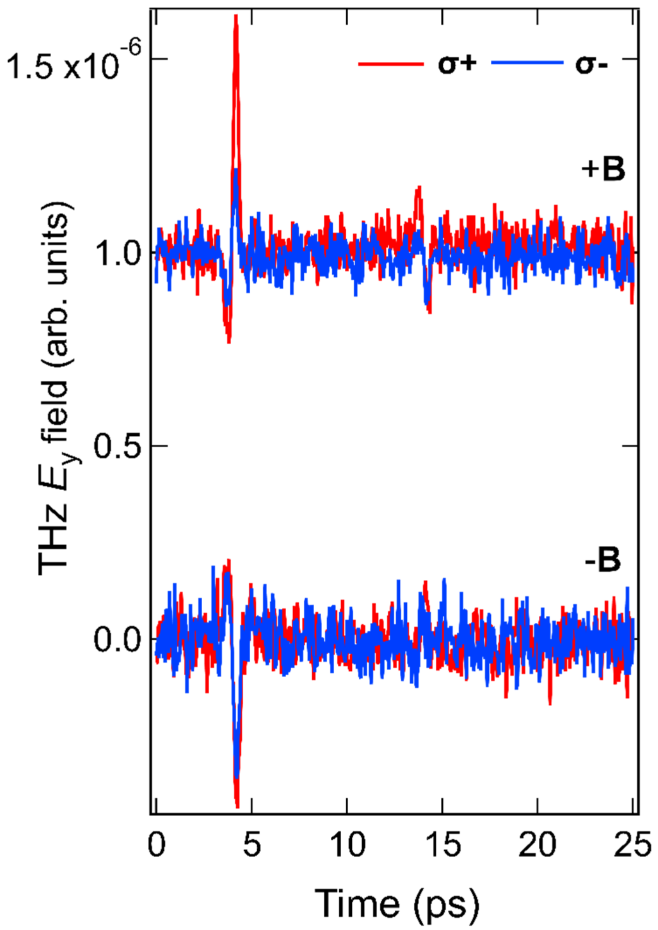

Figure S5. Pump circular polarization dependence for the (n = 1) 2D-HMH/NiFe device. E_y component of THz field demonstrates negligible circular polarization dependence at both fields.

V. THz emission in 3D HMH-based THz emitter

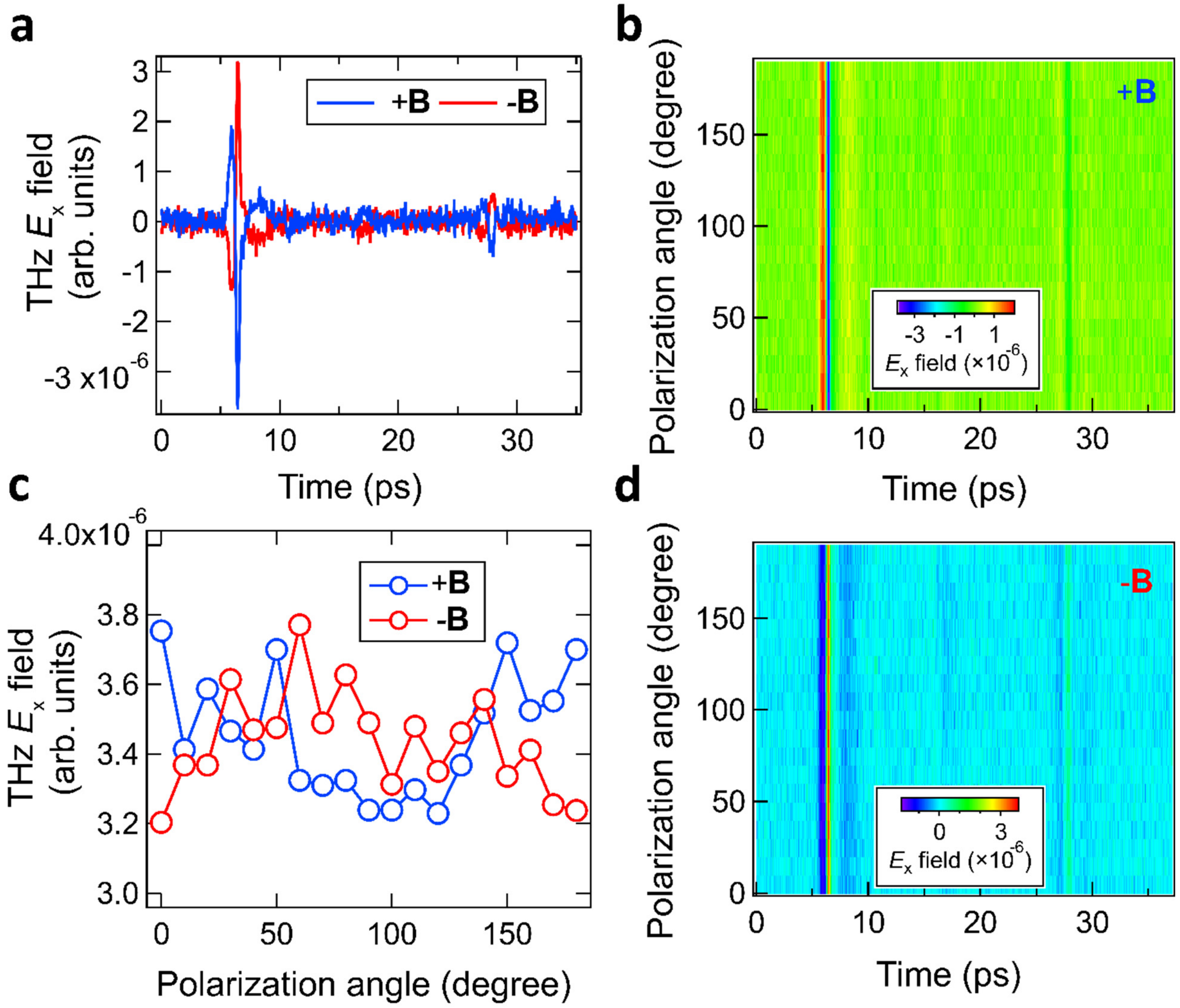

Figure S6. THz emission in 3D-HMH control sample consisting of NiFe/$MAPbBr_3$. **a**, Field dependent THz emission as a function of time. **b** and **d** E_x component of obtained THz field as a function of time and polarization angle of 800 nm optical excitation pulse for +**B** and –**B** field, respectively. **c**, E_x component of the peak THz field as a function of polarization angle for +**B** and –**B** field.

VI. THz transmission

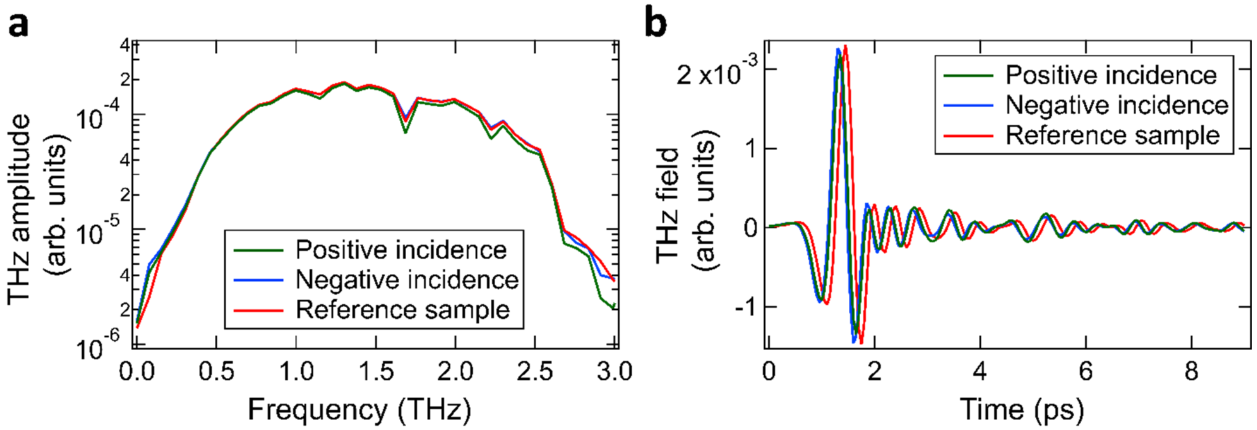

Figure S7. Transmission of THz emission in (n=1) 2D-HMH/NiFe heterostructure and bare substrate reference sample as a function of (**a**) frequency and (**b**) time. The THz pulses are generated from optical rectification of 800 nm pulses from Ti: Sapphire regenerative amplifier (Spectra-Physics Spitfire Ace) in a 0.3 mm-thick GaP crystal. The transmission of the THz through the devices was measured using the same detection scheme as in the THz emission experiment. There is no detectable difference for the THz transmission and absorption along the forward (+*z*) and backward (-*z*) directions.

VII. Sample anisotropy

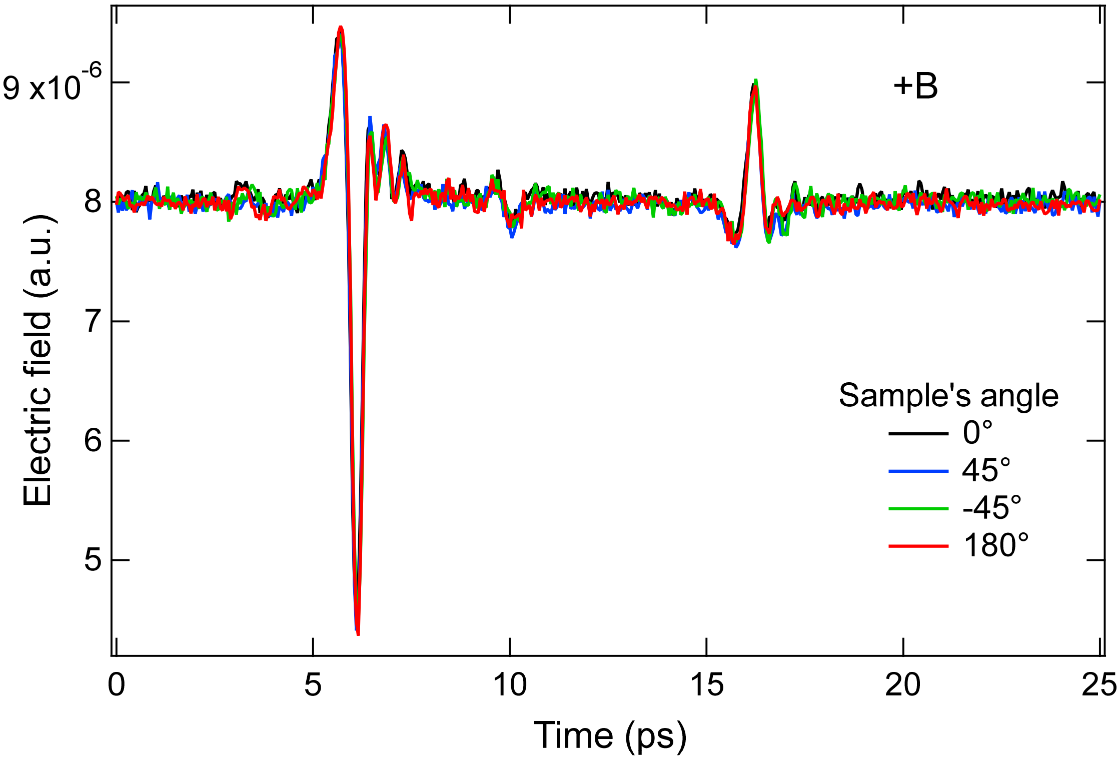

Figure S8. Dependence of E_x component of THz waveform on the sample azimuth rotation angle for (n=1) 2D-HMH sample demonstrating isotropic behavior.

VIII. Role of SiO_2 capping layer for THz emission

It was reported that the Rashba interface can be formed when the ferromagnet layer is interfaced with SiO_2 or Sapphire substrate[1] which can contribute to the generation of the THz emission.

To fully separate the contribution from the possible Rashba interface formed at the SiO_2 or Sapphire substrate[1,2], we have performed a series of spintronic THz experiments in multiple control samples, including Sapphire/SiO_2/NiFe, Sapphire/NiFe/SiO_2, Sapphire/NiFe/Pt, and Sapphire/Pt/NiFe, Sapphire/2D-HMH/NiFe, and Sapphire/3D-HMH/NiFe devices. We measured the THz emission in each device under the same conditions (field strength, laser intensity, sample position, etc.). Following the literature reports, we assumed the formation of the interfacial Rashba/SOC interface between the Py (NiFe) and the Sapphire/SiO_2 substrate (marked as ||) that is responsible for the measured THz electric field. By systematically changing the device stacking configuration, the role of the Rashba effect at the SiO_2 or sapphire interface can be well-separated, from which the actual THz emission from the 2D-HMH/NiFe interface can be derived (see Table S1 below).

We found the THz emission from the potential Rashba interface formed at the Sapphire||Py interface is indeed strong. Its amplitude (84×10^{-6} arb. units) is comparable to that from the Py||Pt interface (146×10^{-6} arb. units). However it is noteworthy in our HMH-based THz configuration, there is no direct contact between Py and Sapphire. Consequently, this interface will not be responsible for the measure THz emission in the 2D-HMH/Py device.

The THz emission from Py||SiO_2 interface is roughly three times weaker (6×10^{-6} arb. units) than that from 2D-HMH||Py interface (22×10^{-6} arb. units). This confirms that the possible Rashba/SOC interface between Py and SiO_2 (if it exists) is not the dominant THz source for the observed signals.

Although the THz intensity from the SiO_2/Py interface does not play a significant role in the observed asymmetric THz emission from the 2D-HMH/Py heterostructures, we indeed observed an asymmetric THz emission as a function of the relative angle (θ) between pump linear polarization axis with respect to the magnetic field, as shown in Fig. S9. However, the change of THz intensity exhibits a different angular dependence ($\propto \sin(2\theta)$) of which the phase is shifted by 90 degrees in contrast that in the 2D-HMH/Py device ($\propto \cos(2\theta)$. Therefore, the

THz emission from SiO_2/Py interface alone cannot explain the observed angular dependence in our sample.

Table S1. Obtained THz intensities in different device structures at the applied positive magnetic field, +B. The THz signal originating from 2D-HMH materials has been calculated. Note: the reversed interface yields opposite THz signal, as confirmed by the reversed THz polarity between the NiFe/Pt and Pt/NiFe device.

Devices (|: normal interface; ||: Rashba/SOC interface)	THz (10^{-6}) from the left interface of Py	THz (10^{-6}) from the right interface of Py	Observed THz field (10^{-6})
Sapphire | SiO_2 || Py	+6	0	+6
Sapphire || Py || SiO_2	-84	-6	-90
**Sapphire | 2D-HMH || Py|| SiO_2**	**+22**	**-6**	**+16**
**Sapphire | 3D-HMH || Py|| SiO_2**	**+10**	**-6**	**+4**
Sapphire || Py || Pt	-84	-146	-230

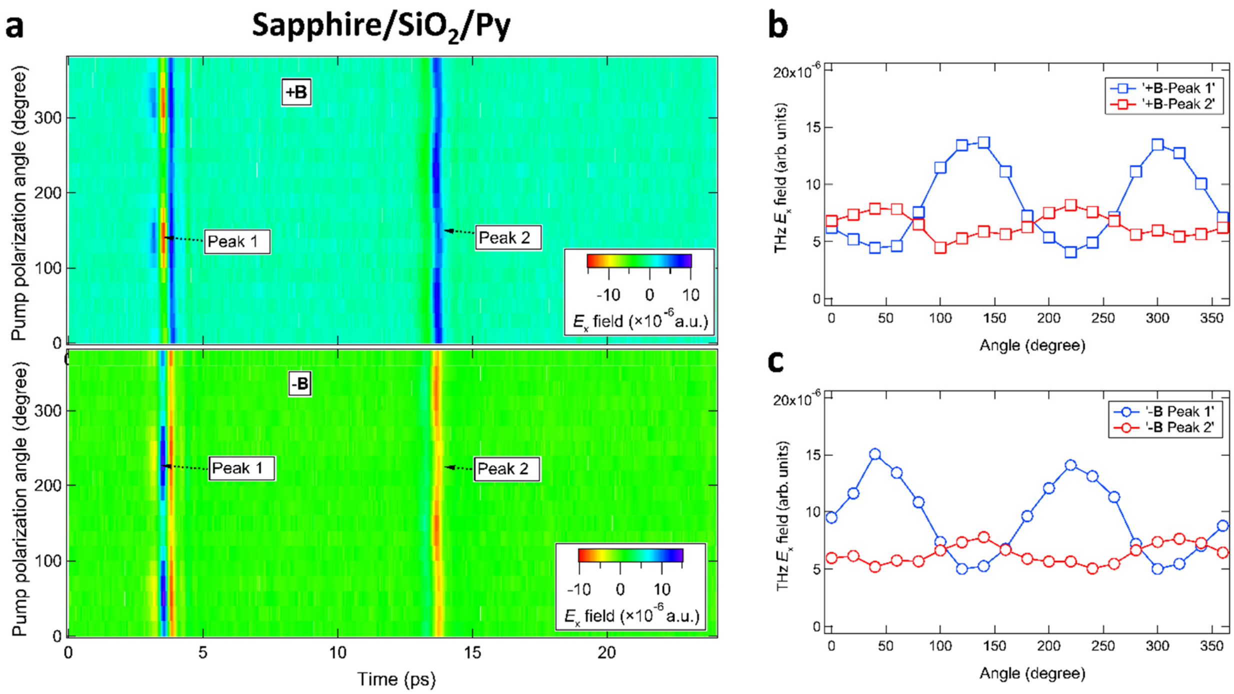

Figure S9. Pump polarization dependence of asymmetric THz radiation in Sapphire/SiO₂/Py sample. **a**, 2D contour plot of the electric field of THz radiation along the *x*-direction as a function of time and polarization angle (θ), exhibiting a linear polarization-modulated THz intensity in both two peak groups. **b** and **c**, summarize the opposite changes of the polarization-dependent THz intensity in Peak 1 and Peak 2 with the switching of the magnetic field, respectively.

IX. Asymmetric THz emission in other reduced-dimensional HMH-based THz emitters

To further validate that the observed asymmetric THz emission was mainly attributed to the 2D-HMH/Py interface, we have fabricated a series of reduced-dimensional HMH-based spintronic THz devices (i.e., $(BA)_2(MA)_{n-1}Pb_nI_{3n+1}/Py/SiO_2$, MA= methylammonium, BA= butylammonium) while maintaining the Py/SiO_2 interface unchanged. Figure S10 shows the obtained magnetic-field-switchable THz emission in thin-film reduced-dimensional HMH-based THz emitters having quantum layers n=1, 2, and 4, respectively. In these devices, the number of inorganic PbI framework increases thus substantially changes the Rashba interface between RD-HMH and Py while the Py/SiO_2 interface remains the same. If the Py/SiO_2 interface dominates the THz signal, the asymmetric feature would be also unchanged. By increasing the quantum layer from n=1 to n=4, we found that the difference of THz intensity between the positive and negative magnetic fields increases with the increasing quantum layer. This suggests the key role of the quantum well effect for the asymmetric THz emission and their correlation with the layer-dependent Rashba state[3], which is confirmed to be separated from the Py/SiO_2 interface.

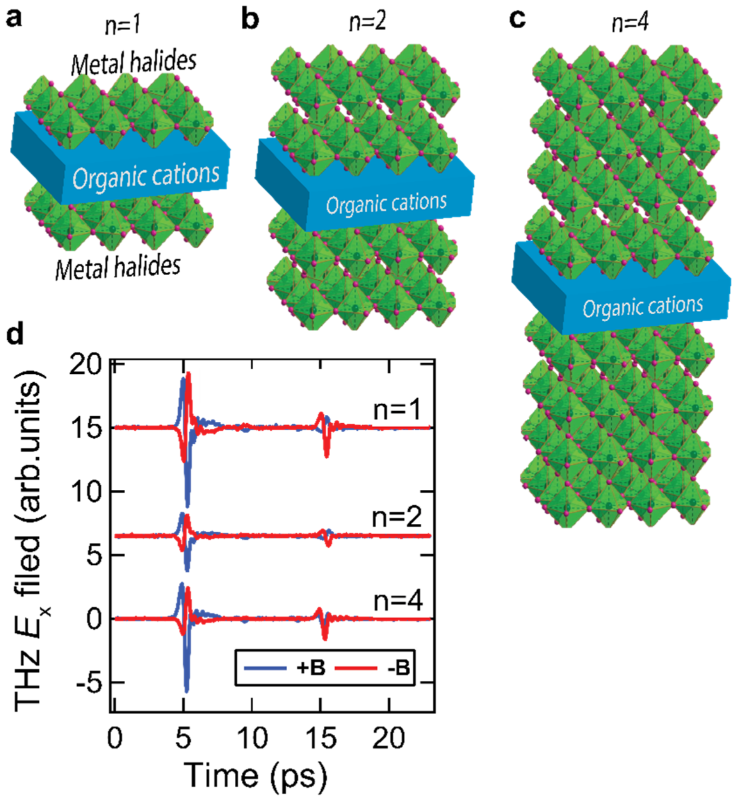

Figure S10. Observations of asymmetric THz emission in $(BA)_2(MA)_{n-1}Pb_nI_{3n+1}$/NiFe devices with the quantum number, n=1, 2, and 4, respectively.

X. Pump fluence dependence

The obtained THz electric field scales nearly linearly at low laser pump fluences (< 0.8 mJ/cm^2), following by a saturating behavior at higher laser fluences. This pronounced sub-linear fluence dependence is consistent with that in Co/Pt THz devices[4], excluding possible high-order effects induced by the laser pulse. It excludes possible heat-driven spin generation in the NiFe layer induced by the laser pulse and resulting THz emission since this scenario would result in a superliner fluence dependence of the THz emission. It is possible that the high laser fluences may induce an unavoidable sample degradation because of the laser absorption in the metallic NiFe layer. This may lead to a change of the THz conductivity in the HMHs owing to their low-thermal conductivities (e.g., 0.5 W·K^{-1}·m^{-1} in $CH_3NH_3PbI_3$)[5], and decreased magnetization of the NiFe layer. Both would be accounted for the saturation behavior of the THz signal at higher laser fluences.

To trade-off between the THz intensity and sample degradation induced by a strong laser pulse, for all our THz measurements, the pump beam with a pump fluence of 1.0 mJ/cm^2 is used to excite the devices in the normal incidence.

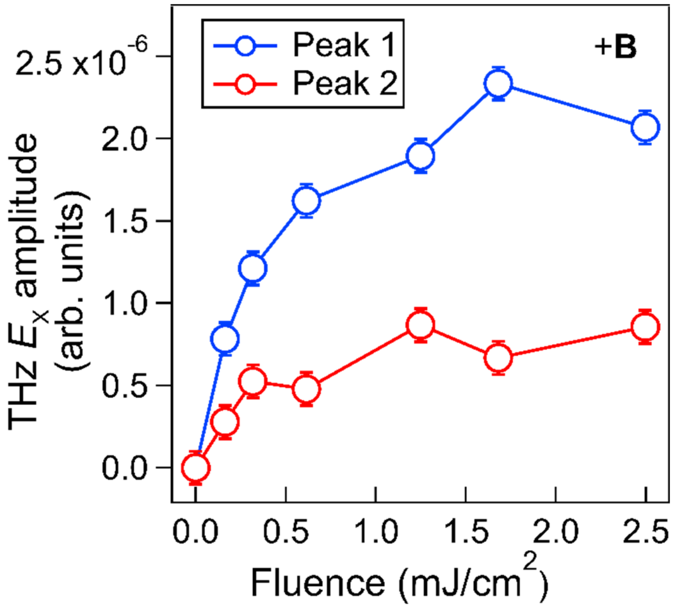

Figure S11. Fluence dependence of the peak-to-peak THz amplitude obtained from the 2D-HMH/NiFe heterostructure. The error bars represent the standard errors of the measurements.

XI. Summary of control experiments

At first glance, the asymmetric THz emission may be intuitively understood as the field-dependent spin-to-charge conversion efficiency (i.e., IREE length) or non-linear optical effect at the layered Rashba state of the 2D-HMH thin film in the presence of an injected superdiffusive spin current. However, the reversed sign of asymmetric THz intensity from the backward THz emission opposes this assumption, corroborated by the equal value of overall THz intensity obtained between two fields as shown in Fig. 2d. The laser/sample misalignment caused by the reversed magnetic field direction have been also excluded by a series of careful control experiments using metallic spintronic THz emitters (S.I. Figs. S4). The field-dependent results manifest the field-controlled asymmetrically distributed THz emission toward +z and -z directions, leading to the asymmetric THz intensity (see the schematic illustration in Fig. 1).

Using the front/back pump configuration (Fig. 3), sample orientation dependence of THz emission further confirms that the hybrid heterostructure-induced spontaneous symmetry breaking does not play a key role in determining the asymmetric THz emission. We also performed helicity-dependent measurements but no sign reversal of the E_y component has been observed (see S.I. Fig. S5). Together with the unchanged THz transmission measurement by flipping the sample (S.I. Fig. S7), and the absence of magnetic anisotropy in the NiFe film (S.I. Fig. S8), sample orientation dependence motivated us to revisit the pump-pulse polarization dependence although it is generally accepted that the linear pump-pulse induced changes in spintronic THz emission are assumed to be negligible.

The realization of coherent control of THz emission up to 40% as shown in Fig. 4 indicates that the generated THz signal as a function of the pump light field, i.e., pump polarization axis, and the relative angle between the polarization axis and magnetic field direction. The absence of asymmetric THz emission and coherent control in the similar 3D-$CH_3NH_3PbBr_3$ materials implies the dominant role of two-dimensionality or the layered structure for the observed phenomena (S.I. Fig. S6).

The role of the SiO_2 capping layer for the asymmetric THz radiation has been carefully conducted as shown in S.I. Fig. S9 and Table S1. By tuning the quantum layer of HMH structure from n=1 to n=4, we confirmed that the difference of THz intensity between the positive and negative magnetic fields increases with the increasing quantum layer (S.I. Fig. S10). This

suggests the key role of the quantum well effect for the asymmetric THz emission, preluding the negligible contribution from the NiFe/SiO_2 interface.

References

1. Luo, Z. *et al.* Spin-Orbit Torque in a Single Ferromagnetic Layer Induced by Surface Spin Rotation. *Phys. Rev. Appl.* **11**, 64021 (2019).

2. Chen, X. *et al.* Giant antidamping orbital torque originating from the orbital Rashba-Edelstein effect in ferromagnetic heterostructures. *Nat. Commun.* **9**, 2569 (2018).

3. Yin, J. *et al.* Layer-Dependent Rashba Band Splitting in 2D Hybrid Perovskites. *Chem. Mater.* **30**, 8538–8545 (2018).

4. Huisman, T. J. *et al.* Femtosecond control of electric currents in metallic ferromagnetic heterostructures. *Nat. Nanotechnol.* **11**, 455–458 (2016).

5. Pisoni, A. *et al.* Ultra-Low Thermal Conductivity in Organic–Inorganic Hybrid Perovskite CH3NH3PbI3. *J. Phys. Chem. Lett.* **5**, 2488–2492 (2014).
